# The Influence of *CYP3A4* Polymorphism in Sex Steroids as a Risk Factor for Breast Cancer

**DOI:** 10.1055/s-0038-1673365

**Published:** 2018-10-18

**Authors:** Melissa Gonzalez Veiga, Rogério Tadeu Felizi, Dayane Guerino Reis, Ivo Carelli Filho, Cesar Eduardo Fernandes, Ricardo Peres do Souto, Emerson Oliveira

**Affiliations:** 1 Department of Gynecology, Faculty of Medicine of ABC, Santo André, SP, Brazil; 2 Department of Biochemistry, Faculty of Medicine of ABC, Santo André, SP, Brazil

**Keywords:** Cyp3a4, breast cancer, polymorphism, estrogens, genetics, CYP3A4, câncer de mama, polimorfismo, estrógenos, genética

## Abstract

**Objective** Epidemiological studies have shown evidence of the effect of sex hormones in the pathogenesis of breast cancer, and have suggested a relationship of the disease with variations in genes involved in estrogen synthesis and/or metabolism. The aim of the present study was to evaluate the association between the CYP3A4*1B gene polymorphism (rs2740574) and the risk of developing breast cancer.

**Methods** In the present case-control study, the frequency of the CYP3A4*1B gene polymorphism was determined in 148 women with breast cancer and in 245 women without the disease. The DNA of the participants was extracted from plasma samples, and the gene was amplified by polymerase chain reaction. The presence of the polymorphism was determined using restriction enzymes.

**Results** After adjusting for confounding variables, we have found that the polymorphism was not associated with the occurrence of breast cancer (odds ratio = 1.151; 95% confidence interval: 0.714–1.856; *p* = 0.564). We have also found no association with the presence of hormone receptors, with human epidermal growth factor receptor 2 (HER2) overexpression, or with the rate of tumor cell proliferation.

**Conclusion** We have not observed a relationship between the CYP3A4*1B gene polymorphism and the occurrence of breast cancer.

## Abstract (pt)

**Resumo:**

**Objetivo** Estudos epidemiológicos têm mostrado evidências da influência dos hormônios sexuais na patogênese do câncer de mama, e têm sugerido uma relação entre a doença e variações em genes envolvidos na síntese e/ou metabolização de estrógenos. O objetivo do presente estudo foi avaliar a associação entre o polimorfismo do gene CYP3A4*1B (rs2740574) e o risco de desenvolvimento da neoplasia mamária.

**Métodos** No presente estudo de caso-controle, a frequência de polimorfismo do gene CYP3A4*1B foi determinada em 148 mulheres com câncer de mama, e em 245 mulheres sem a doença. O DNA das participantes foi extraído do plasma, e o gene foi amplificado por meio de reação em cadeia da polimerase, enquanto o polimorfismo foi determinado por enzimas de restrição.

**Resultados** O polimorfismo, após o ajuste para variáveis de confusão, não foi associado à ocorrência de câncer de mama (razão de possibilidades = 1,151; intervalo de confiança de 95%: 0,714–1,856; *p* = 0,564). Também não observamos associação com a presença de receptores hormonais, superexpressão do receptor tipo 2 do fator de crescimento epidérmico humano (HER2, na sigla em inglês), ou com a taxa de proliferação celular do tumor.

**Conclusão** Não observamos relação entre o polimorfismo do gene CYP3A4*1B e a ocorrência de câncer de mama.

## Introduction

Breast cancer is the most common type of cancer in the female population, second only to cases of non-melanoma skin cancer. The mortality rate due to the disease presents an upward curve,^1^ contributing to make breast cancer a major public health problem and an important cause of mortality in adults.^2^ In 2018, 59,700 new cases were estimated in Brazil, representing an incidence rate of more than 56 cases per 100,000 women.^1^ A previous family history of the disease is present in ∼ 10 to 15% of the breast cancer patients. However, only 5% of the cases can be explained by mutation of genes such as *BRCA1* and *BRCA2*.^3^ Regarding the family risk for the development of the disease, it is necessary to consider the influence of environmental factors and genetic variations that may alter the predisposition to the risk of breast cancer.^4^

CYP3A4 is an enzyme of the cytochrome P450 family, encoded by the *CYP3A4* gene, which plays a key role in the metabolism of estrogens, catalyzing its hydroxylation in the liver; it contributes with other enzymes that also participate in this process, both intrahepatically and extrahepatically. In the hydroxylation process catalyzed by these enzymes, estradiol is converted to 2-hydroxyoestradiol, a hormone metabolite that has a low carcinogenic potential.^5^

Several studies have shown that exposure to estrogen plays an important role in the etiology of breast cancer.^6^ ^7^ Because estrogens and their metabolites are known as inducers and promoters of tumor growth, genes encoding enzymes involved in their metabolism are hypothetically involved in the pathogenesis of this neoplasm.^8^ ^9^

Recently, numerous researchers have focused their studies on some gene polymorphisms of estrogen metabolism and, apparently, the influence of these changes on the risk of developing breast cancer is low. However, as these are common changes, it is plausible that they may be responsible for a large number of cases of the disease.^10^

Of the many single nucleotide polymorphisms (SNPs) that have been identified in the *CYP3A4* gene, the CYP3A4*1B variant is one of the most common polymorphisms, and has been associated with specific types of cancer, including breast cancer.^2^ The CYP3A4*1B polymorphism (rs2740574) corresponds to an A to G substitution at the position -290 of the gene promoter, which results in a lower expression of CYP3A4 or a decrease in the catalytic activity of the enzyme.^11^ Some studies have evaluated the polymorphism in question with regards to the predisposition to breast cancer, without an association being clearly established.^12^ ^13^ ^14^ ^15^ ^16^

In the present clinical, cross-sectional case-control study, we have evaluated the potential relationship of the *CYP3A4* gene polymorphism with breast cancer.

## Methods

We studied 393 women recruited between 2013 and 2015, who were followed-up in the Mastology Sector of the Division of Gynecology of Faculdade de Medicina do ABC (FMABC, in the Portuguese acronym). The project was approved by the Ethics in Research Committee of the institution under the number 169/2010. The participants were divided into 2 groups: 148 women with a histologically confirmed diagnosis of breast cancer (case group), and 245 women without the disease, with normal clinical and mammographic examinations (control group). For the patients with breast cancer, an immunohistochemical analysis of the tumor was performed to determine the presence of estrogen receptors, detected using the EP1 clone. Clinical data were collected with the use of a questionnaire. The following data were recorded: age, age at menarche and last menstruation, number of pregnancies, previous use of hormonal medications, breastfeeding, history of smoking, alcohol consumption, and endocrine diseases. The patients included were informed about the study and signed a consent form.

**Fig. 1:**
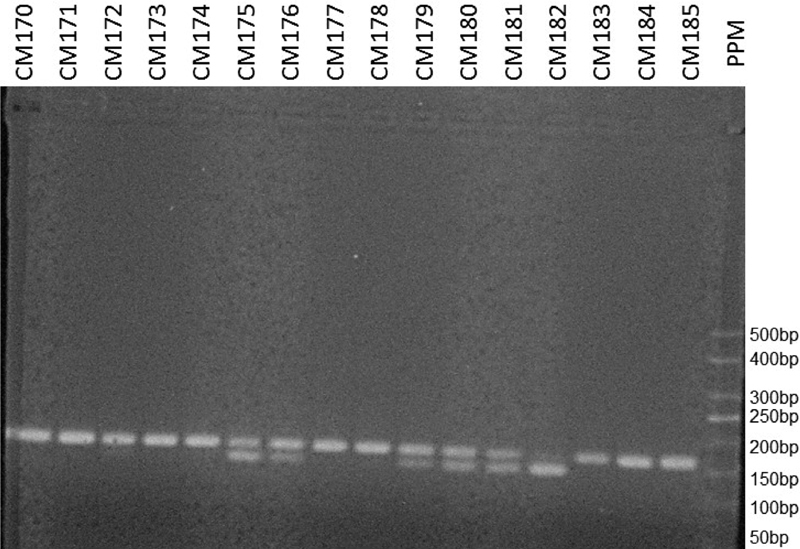
Polymerase chain reaction products visualized on ethidium bromide-stained 3% agarose gels.

Venous blood samples were collected from the women in both groups, and the genomic DNA was extracted using the Illustra blood genomic prep mini spin reagent kit (GE Healthcare Life Sciences, Buckinghamshire, UK)), following the manufacturer's instructions. The presence of the *CYP3A4* gene polymorphism was determined following the polymerase chain reaction restriction fragment length polymorphism (PCR-RFLP) procedure described by Voso et al.^17^ For the amplification of the promoter region of the gene by polymerase chain reaction (PCR), the following primers were used: 5′GGA CAG CCA TAG AGA CAA GGG CC-3' and 5′TCA CTG ACC TCC TTT GAG TTC ATA-3′. The 165-bp PCR products were treated with the *MspI* restriction enzyme, and the restriction fragments were separated by electrophoresis in 3.0% agarose stained with ethidium bromide. At the end of the analysis, A/A homozygotes should present a single 165-bp band, G/G homozygotes should present 2 bands of 142 and 23 bp, and A/G heterozygotes should present 3 bands of 165, 142 and 23 bp (Fig. 1).

To assess the association between the study groups and the categorical variables, we have used the frequency chi-squared test, whereas the continuous variables were analyzed using the unpaired *t*-test. The Hardy-Weinberg equilibrium was also tested using the chi-squared test. After the stratification of the groups, the effect of the *CYP3A4* gene polymorphism on breast cancer development was estimated by the odds ratio (OR), obtained by the binary logistic regression model, using IBM SPSS Statistics for Windows, version 23.0 (IBM Corp, Armonk, NY, US). The confidence interval (CI) adopted was 95%, and the value for rejection of the null hypothesis was set at 0.05 or 5% (α ≤ 0.05).

## Results

The clinical and epidemiological characteristics of the case and control groups are described in Table 1. Both groups presented homogeneity for almost all of the characteristics evaluated, with similar proportions of women > 50 years old, of menopausal women and/or of women who used hormone therapy. The variable parity and the age at first pregnancy also showed no significant differences between the groups. The cases were more likely to use oral contraceptives than the controls, with the frequency of use at 22.3% and 6.1% respectively (*p* < 0.0001). The family history of breast cancer (*p* = 0.04) was more frequent in women who presented with the disease, with a difference of almost 10% between the groups.

**Table 1:** Clinical characteristics of cases and controls

Characteristics	Cases (*n* = 148)	Controls (*n* = 245)	*p*-value
Age (years)^#^	57.8 ± 0.9	59.5 ± 0.6	0.134
Age at menarche (years)^#^	12.9 ± 0.1	13.2 ± 0.1	0.059
Postmenopause*	121 (81.7%)	82.7%	0.785
Parity*	2.6 ± 0.12	2.9 ± 0.09	0.067
Breastfeeding*	116 (78.4%)	207 (84.5%)	0.13
Age at first pregnancy^#^	23.1 ± 0.45	22.7 ± 0.3	0.54
Use of oral contraceptive*	33 (22.3%)	15 (6.1%)	< 0.0001**
Use of hormone therapy*	13 (8.7%)	36 (14.7%)	0.114
Family history of breast cancer*	25 (16.9%)	18 (7.3%)	0.004**

Note: Continuous variables: values expressed as the mean and standard deviation; categorical variables: values expressed as numbers and percentages; ^#^unpaired *t*-test; *chi-squared test; **significant values.

The genotyping and the frequency of the alleles are described in Table 2.

**Table 2:** CPYP3A4*1B polymorphism and occurrence of breast cancer

	AA	AG + GG	OR crude (CI)	OR adjusted (CI)*
Cases	76	72	1.69 (1.116–2.559) *p* = 0.013	1.151 (0.714–1.856) *p* = 0.564
Controls	157	88		

Abbreviations: CI, confidence interval; OR, odds ratio.

Note: *Values adjusted for the use of oral contraceptives and family history of breast cancer.

Due to the low incidence of the GG genotype in the studied population, we have chosen to analyze the results comparing the wild homozygous group (AA) with the polymorphic group (AG + GG). After adjusting for oral contraceptive use and family history of breast cancer, the presence of the G allele and the GG (AG + GG) genotype of the CYP3A4*1B polymorphism was not directly associated with tumor occurrence (OR = 1.151; 95%CI: 0.714–1.856; *p* = 0.564). In addition, no statistically significant difference was found between the polymorphisms when they were analyzed according to the estrogen receptor status, to human epidermal growth factor receptor 2 (HER2) overexpression or non-overexpression, or to cell proliferation rate represented by Ki67, as shown in Table 3.

**Table 3:** Evaluation of the CYP3A4*1 *B* polymorphism and status of estrogen receptor, HER2 and Ki67

	AA, n (%)	AG + GG, n (%)	*p*-value*
Estrogen receptor +	54 (48.2%)	58 (51.8%)	0.186
Estrogen receptor -	22 (61.1%)	14 (38.9%)	
HER2 +	11 (45.8%)	13 (54.2%)	0.65
HER2 -	65 (52.4%)	59 (47.6%)	
Ki67 ≤ 25%	41 (58.6%)	29 (41.4%)	0.158
Ki67 > 25%	27 (45%)	33 (55%)	

Abbreviation: HER2, human epidermal growth factor receptor 2.

Note: *Chi-squared test.

## Discussion

The distribution of the genotypes is not in genetic equilibrium according to the Hardy-Weinberg principle, which has also been observed in some of the previous studies that evaluated the same polymorphism. This fact can be explained by the excess of the CYP3A4*1B gene homozygous variant or by the high frequency of the wild variant when compared with that of the polymorphism, although this hypothesis has not been clearly discussed in the literature.^2^ ^18^ ^19^ ^20^ ^21^ ^22^ Hereditary predisposition to breast cancer significantly influences the screening and follow-up of women at high risk of developing the disease. However, in patients with a personal or family history of breast cancer, a specific genetic predisposition is identified in less than 30% of the cases.^23^ Thus, it seems that the effect of low penetrance gene polymorphisms on the risk for breast cancer is relevant only in polygenic forms.^23^

Genetic factors have been described as modifiers of estrogen levels and good candidates for breast cancer predisposition alleles.^12^ Genetic variations found in the *CYP3A4* gene, located in the chromosome 7q21.3-q22.1, may influence the level or function of the CYP3A4 protein.^2^ Single nucleotide polymorphisms have already been identified in the *CYP3A4* gene, and the most common variant is the CYP3A4*1B gene, an A290G substitution in the 5′ flanking region.^24^ The CYP3A4*1B gene polymorphism was hypothesized to cause reduced *CYP3A4* gene expression.^16^ Our study demonstrated that the G allele and the GG genotype of the CYP3A4*1B gene polymorphism were not directly associated with the occurrence of breast cancer, as shown in table 2.

The association between this polymorphism and the disease has already been studied by groups from several countries, without a direct relationship being established. A Chilean study found a higher frequency of the polymorphism in patients with breast cancer when compared with healthy women, although the difference was not statistically significant (OR = 1.83; *p* = 0.212).^25^ In 1998, a prospective study involving more than 2,700 women also evaluated the relationship between breast cancer and the CYP3A4*1B gene, and found no association.^10^ Similarly, an Australian study also found no association between breast cancer and the CYP3A4*1B gene, even when the outcome was adjusted for age and menopausal status (OR = 0.86; 95%CI: 0.54 - 1.33).^16^ In addition, a 2012 large systematic review followed by a meta-analysis, which included 11 studies and nearly 7,000 patients, did not find any evidence that the CYP3A4*1B gene is related to the risk of cancer.^2^

Genetic variations in enzymes involved in steroidogenesis have been suggested to play a role not only in the risk of breast cancer, but also in the age at menarche.^25^ The association of earlier menarche with the presence of the CYP3A4*1B gene has been demonstrated in a study conducted with women from the United States (adjusted OR = 3.21; 95% CI: 1.62–6.89).^25^

The possible relationship between the polymorphism in question and breast cancer was suggested by Kadlubar et al^26^ due to the positive association found between the polymorphic variant and the age at menarche, a recognized risk factor for the development of the disease.^25^ In our study, the age at menarche was lower in the case group than in the control group (*p* = 0.059).

A factor with strong involvement that has not yet been established as a risk factor is the use of oral contraceptives, which, in our study, was related to a higher incidence of the disease (*p* < 0.0001).^27^ A meta-analysis correlating Iranian studies demonstrated that the use of oral contraceptives may stimulate the occurrence of breast cancer because it directly increases estrogen levels and indirectly influences weight gain.^28^ In a recent prospective cohort study, a relative risk of breast cancer of 1.20 was found (95%CI: 1.14–1.26) among users of hormonal contraception, as compared with women who had never used hormonal contraception.^29^ Our finding is also consistent with the results reported in an analysis published in 2016 that showed an OR of breast cancer development that was 54.6% lower in patients who did not use oral contraceptives compared with those who used them.^30^

Approximately 5 to 10% of breast cancer cases are familial and occur earlier than those in the general population. The BRCA1 and BRCA2 mutations are primarily responsible for hereditary breast cancer.^31^ Despite years of research, it has been shown that a minority of patients with a personal or family history of breast cancer have a genetic mutation as an identifiable cause.^23^ The present study is consistent with the global literature, as we have found a positive association of family history with the development of the disease (*p* = 0.004).

A stratified analysis according to HER2 or to estrogen receptor expression in neoplastic cells showed no relationship with the occurrence of the polymorphism studied. Similarly, Ki67–a tumor cell proliferation index –was not a factor associated with the greater presence of polymorphic alleles. We believe, however, that more studies are needed to confirm any of the proposed hypotheses due to the lack of evidence in the literature on the subject.

We note that the controversy remains over the influence of the CYP3A4*1B gene on the genesis of breast cancer. More studies and a larger case sample are necessary to confirm the effects on the risk of breast cancer to assist in the screening and follow-up of patients at increased risk of the disease.

The main results of the present study suggest that the G allele and the GG genotype of the CYP3A4*1B gene do not play a key role in breast cancer development.

The small sample size and the breast cancer risk factors were among the limitations of the present study that might have affected the detection of differences between the groups.

## Conclusion

We did not observe a relationship between the CYP3A4*1B gene polymorphism and the occurrence of breast cancer.

## References

[1] Ministério da Saúde. Instituto Nacional de Câncer José Alencar Gomes da Silva. *Estimativa 2018: Incidência de Câncer no Brasil.* Rio de Janeiro, RJ: INCA; 2017 http://www.inca.gov.br/estimativa/2018/estimativa-2018.pdf. Accessed December 17, 2017.

[2] Zhou L P Yao F Luan H CYP3A4*1B polymorphism and cancer risk: a HuGE review and meta-analysis Tumour Biol 2013 34 02 649 660 Doi: 10.1007/s13277-012-0592-z 23179402 10.1007/s13277-012-0592-z

[3] Newman B Austin M A Lee M King M C Inheritance of human breast cancer: evidence for autosomal dominant transmission in high-risk families Proc Natl Acad Sci U S A 1988 85 09 3044 3048 Doi: 10.1073/pnas.85.9.3044 3362861 10.1073/pnas.85.9.3044 PMC280139

[4] Dunning A M Healey C S Pharoah P DP Teare M D Ponder B A Easton D F A systematic review of genetic polymorphisms and breast cancer risk Cancer Epidemiol Biomarkers Prev 1999 8 10 843 854 10548311

[5] Tsuchiya Y Nakajima M Yokoi T Cytochrome P450-mediated metabolism of estrogens and its regulation in human Cancer Lett 2005 227 02 115 124 Doi: 10.1016/j.canlet.2004.10.007 PubMed 16112414 10.1016/j.canlet.2004.10.007

[6] Feigelson H S Breast cancer: epidemiology and molecular endocrinology New York, NY Oxford University Press 2003 120 138

[7] Thomas H V Reeves G K Key T J Endogenous estrogen and postmenopausal breast cancer: a quantitative review Cancer Causes Control 1997 8 06 922 928 9427435 10.1023/a:1018476631561

[8] Hefler L A Tempfer C B Grimm C Estrogen-metabolizing gene polymorphisms in the assessment of breast carcinoma risk and fibroadenoma risk in Caucasian women Cancer 2004 101 02 264 269 Doi: 10.1002/cncr.20361 15241822 10.1002/cncr.20361

[9] Service R F New role for estrogen in cancer? Science 1998 279(5357):1631 1633 Doi: 10.1126/science.279.5357.1631 9518374 10.1126/science.279.5357.1631

[10] Le Marchand L Donlon T Kolonel L N Henderson B E Wilkens L R Estrogen metabolism-related genes and breast cancer risk: the multiethnic cohort study Cancer Epidemiol Biomarkers Prev 2005 14 08 1998 2003 Doi: 10.1158/1055-9965.EPI-05-0076 16103451 10.1158/1055-9965.EPI-05-0076

[11] Dally H Edler L Jäger B The CYP3A4*1B allele increases risk for small cell lung cancer: effect of gender and smoking dose Pharmacogenetics 2003 13 10 607 618 Doi: 10.1097/01.fpc.0000054128.14659.a6 14515059 10.1097/00008571-200310000-00004

[12] Johnson N Walker K Gibson L J CYP3A variation, premenopausal estrone levels, and breast cancer risk J Natl Cancer Inst 2012 104 09 657 669 Doi: 10.1093/jnci/djs156 22472546 10.1093/jnci/djs156

[13] Rebbeck T R Troxel A B Shatalova E G Lack of effect modification between estrogen metabolism genotypes and combined hormone replacement therapy in postmenopausal breast cancer risk Cancer Epidemiol Biomarkers Prev 2007 16 06 1318 1320 Doi: 10.1158/1055-9965.EPI-07-0084 17548708 10.1158/1055-9965.EPI-07-0084

[14] MARIE-GENICA Consortium on Genetic Susceptibility for Menopausal Hormone Therapy Related Breast Cancer Risk. Genetic polymorphisms in phase I and phase II enzymes and breast cancer risk associated with menopausal hormone therapy in postmenopausal women Breast Cancer Res Treat 2010 119 02 463 474 Doi: 10.1007/s10549-009-0407-0 19424794 10.1007/s10549-009-0407-0

[15] Kato I Cichon M Yee C L Land S Korczak J F African American-preponderant single nucleotide polymorphisms (SNPs) and risk of breast cancer Cancer Epidemiol 2009 33 01 24 30 Doi: 10.1016/j.canep.2009.04.009 19679043 10.1016/j.canep.2009.04.009 PMC2761149

[16] Spurdle A B Goodwin B Hodgson E The CYP3A4*1B polymorphism has no functional significance and is not associated with risk of breast or ovarian cancer Pharmacogenetics 2002 12 05 355 366 12142725 10.1097/00008571-200207000-00003

[17] Voso M T Fabiani E D'Alo' F Increased risk of acute myeloid leukaemia due to polymorphisms in detoxification and DNA repair enzymes Ann Oncol 2007 18 09 1523 1528 Doi: 10.1093/annonc/mdm191 17761709 10.1093/annonc/mdm191

[18] Rebbeck T R Jaffe J M Walker A H Wein A J Malkowicz S B Modification of clinical presentation of prostate tumors by a novel genetic variant in CYP3A4 J Natl Cancer Inst 1998 90 16 1225 1229 Doi: 10.1093/jnci/90.16.1225 9719084 10.1093/jnci/90.16.1225

[19] Walker A H Jaffe J M Gunasegaram S Characterization of an allelic variant in the nifedipine-specific element of CYP3A4: ethnic distribution and implications for prostate cancer risk. Mutations in brief no. 191. Online Hum Mutat 1998 12 04 289 10660343

[20] Paris P L Kupelian P A Hall J M Association between a CYP3A4 genetic variant and clinical presentation in African-American prostate cancer patients Cancer Epidemiol Biomarkers Prev 1999 8 10 901 905 10548319

[21] García-Martín E Martínez C Pizarro R M CYP3A4 variant alleles in white individuals with low CYP3A4 enzyme activity Clin Pharmacol Ther 2002 71 03 196 204 Doi: 10.1067/mcp.2002.121371 11907494 10.1067/mcp.2002.121371

[22] Zeigler-Johnson C M Walker A H Mancke B Ethnic differences in the frequency of prostate cancer susceptibility alleles at SRD5A2 and CYP3A4 Hum Hered 2002 54 01 13 21 Doi: 10.1159/000066695 12446983 10.1159/000066695

[23] Shiovitz S Korde L A Genetics of breast cancer: a topic in evolution Ann Oncol 2015 26 07 1291 1299 Doi: 10.1093/annonc/mdv022 25605744 10.1093/annonc/mdv022 PMC4478970

[24] Lee S J Goldstein J A Functionally defective or altered CYP3A4 and CYP3A5 single nucleotide polymorphisms and their detection with genotyping tests Pharmacogenomics 2005 6 04 357 371 16004554 10.1517/14622416.6.4.357

[25] Fleitas B L Durán M N Miranda M C Lee C K Quiñones S L Estudio de polimorfismos genéticos en CYP3A4 y CYP2D6, y su papel en la susceptibilidad a cáncer de mama Rev Hosp Clin Univ Chile. 2013 24 95 104

[26] Kadlubar F F Berkowitz G S Delongchamp R R The CYP3A4*1B variant is related to the onset of puberty, a known risk factor for the development of breast cancer Cancer Epidemiol Biomarkers Prev 2003 12 04 327 331 12692107

[27] Key T J Verkasalo P K Banks E Epidemiology of breast cancer Lancet Oncol 2001 2 03 133 140 Doi: 10.1016/S1470-2045(00)00254-0 11902563 10.1016/S1470-2045(00)00254-0

[28] Soroush A Farshchian N Komasi S Izadi N Amirifard N Shahmohammadi A The role of oral contraceptive pills on increased risk of breast cancer in Iranian populations: a meta-analysis J Cancer Prev 2016 21 04 294 301 Doi: 10.15430/JCP.2016.21.4.294 28053965 10.15430/JCP.2016.21.4.294 PMC5207615

[29] Mørch L S Skovlund C W Hannaford P C Iversen L Fielding S Lidegaard Ø Contemporary hormonal contraception and the risk of breast cancer N Engl J Med 2017 377 23 2228 2239 Doi: 10.1056/NEJMoa1700732 29211679 10.1056/NEJMoa1700732

[30] Cauchi J P Camilleri L Scerri C Environmental and lifestyle risk factors of breast cancer in Malta-a retrospective case-control study EPMA J 2016 7 20 Doi: 10.1186/s13167-016-0069-z 27679672 10.1186/s13167-016-0069-z PMC5029064

[31] Appel S J Cleiment R J Identifying women at risk for hereditary breast and ovarian cancer syndrome utilizing breast care nurse navigation at mammography and imaging centers J Natl Black Nurses Assoc 2015 26 02 17 26 27045154

